# Target fishing: from “needle in haystack” to “precise guidance”--new technology, new strategy and new opportunity

**DOI:** 10.3389/fphar.2025.1673688

**Published:** 2025-11-07

**Authors:** Juan Chen, Yafei Guo, Jing Shao, Mei Guo, Xinyu Zhu

**Affiliations:** 1 College of Pharmacy, Gansu University of Chinese Medicine, Lanzhou, Gansu, China; 2 School of Traditional Chinese Medicine, Capital Medical University, Beijing, China

**Keywords:** machine learning, artificial intelligence, target fishing, nature products, drug discovery

## Abstract

Drug target discovery is the core breakthrough point of new drug research and development. The chemical complexity and biological network regulation characteristics of natural product systems with a long history of clinical application pose a challenge to the traditional single-target research paradigm. Although traditional technologies based on molecular docking and chemical probes are still dominant, breakthroughs in disruptive technologies such as artificial intelligence and deep learning are driving the transformation of research methods from ‘broad-spectrum screening’ to ‘precise capture’. This review systematically discusses the latest progress of drug target capture technology. Studies have shown that the deep integration of deep learning and knowledge graph not only significantly improves the accuracy of target prediction, but also constructs an interdisciplinary collaboration network across chemical informatics, systems biology and clinical medicine. The fusion of this technology shows three core advantages: multi-dimensional drug-target interaction analysis ability based on deep representation learning; integrate the dynamic predictive modeling ability of multi-omics data; and the interpretable decision support ability with clinical transformability. The purpose of this paper is to provide a theoretical framework for the academic community, and to build a bridge from basic research to clinical application, so as to promote the development of precision drugs into a new era of intelligent drive.

## 1 Introduction

In the progression of human disease treatment, a central challenge in drug discovery lies in the precise identification and validation of molecular targets that can modulate disease pathways. Historically, the field has relied on conventional strategies such as phenotypic screening (Ege et al., 2021), genomics analysis (Yang et al., 2024) and chemical genetics approaches (Arang et al., 2023). Although these approaches have successfully facilitated the discovery of several critical targets—exemplified by the identification of p60AmotL2 by Fonseca et al. and the investigation into *Mycobacterium tuberculosis* targets by Li et al—their overall efficiency remains limited (Li et al., 2022; Fonseca et al., 2024). Inherent limitations, including low screening throughput and protracted timelines for target validation, often result in a vast number of potential targets remaining obscured within the complexity of biological systems (Gu et al., 2021).

**FIGURE 1:**
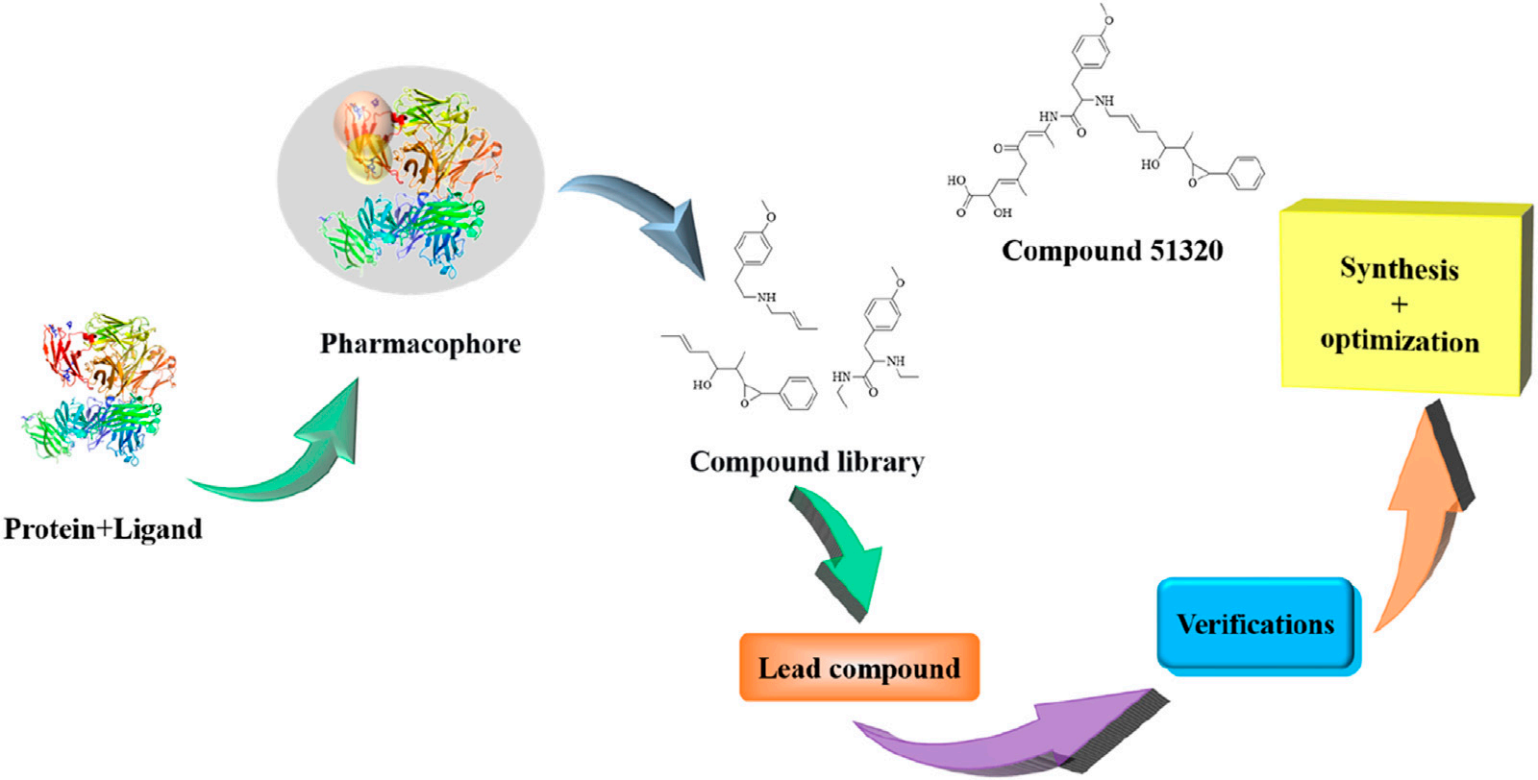
The find of compound 51320.

To address these limitations, innovative research strategies represented by “target fishing” have emerged in recent years. This approach integrates chemical biology, high-resolution proteomics, and artificial intelligence technologies, driving drug discovery from an experience-oriented paradigm toward a data-driven one. Its core mechanism involves using active small molecules as probes to directly “fish” for binding proteins from complex biological samples, thereby reversing the conventional research path from “target-to-drug” to “drug-to-target” (Moumbock et al., 2019). Currently, multiple mature technical frameworks have been established based on different principles, including ligand-based, receptor-based, and complex structure-based methods. The efficacy of this strategy has been demonstrated in numerous studies, such as the structural optimization of berberine by Bin Hong’s team, the discovery of a PD-L1 inhibitor by Luo et al., (Figure 1) and the elucidation of the mechanism of action of celastrol by Wang Jigang’s team, all of which validate the distinct advantages of “target fishing” in target identification and mechanistic exploration (Luo et al., 2021).

**FIGURE 2:**
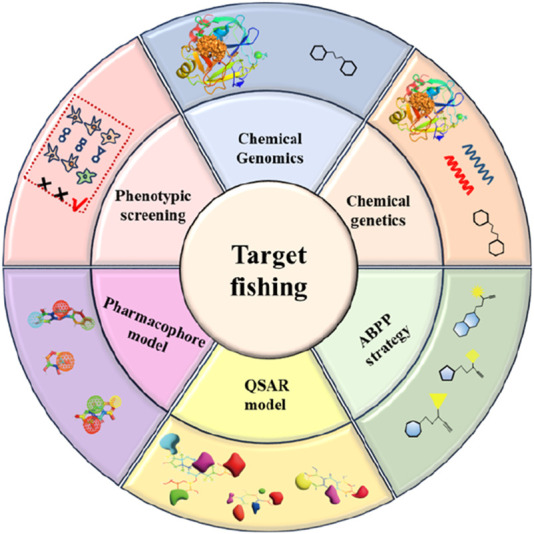
Examples of target fishing methods.

Despite the rapid advancement of “target fishing” technologies and existing reviews that have synthesized their methodologies and application cases, a significant gap remains in the current field: most available summaries focus primarily on technical principles or specific application scenarios, while a systematic integration and critical evaluation of the drug-active ingredients and their corresponding targets discovered through this approach is still lacking. To address this gap, this review aims to systematically summarize the active compounds and their biological targets identified via the “target fishing” strategy, analyze the core distinctions and potential advantages of this technology over conventional methods, and outline future research directions integrating artificial intelligence (Figure 2). We anticipate that this work will provide researchers in the field with a systematic and forward-looking academic reference.

## 2 Ligand-based to screen drug targets

Target fishing uses ligand-based approaches to identify drug targets by analyzing the similarities between active molecules in pharmaceuticals and the corresponding chemicals associated with known disease targets. The primary methodologies utilized in this context include the pharmacophore model and quantitative structure-activity relationship (QSAR) analysis (Galati et al., 2021). At the same time, the combination of multi-ligand fishing technology with various highly sensitive instruments facilitates the rapid identification and purification of natural products (Miranda De Souza Duarte-Filho et al., 2023). 2D and 3D molecular descriptors are ligand fishing techniques developed based on the principle that compounds with similar chemical structures have the same biological activity. Among them, 2D descriptors are dominant in the discovery of ligands, while 3D molecular descriptors have unique advantages in the verification of biological activity (Du et al., 2023). The techniques discussed herein represent the predominant strategies employed in contemporary drug research and development. For instance, in a model of Cisplatin-induced Acute Kidney Injury (AKI) in mice, alisol B has been shown to reduce apoptosis by modulating the Bax/Bcl-2 ratio through the p53 signaling pathway. Additionally, it mitigates renal inflammation by inhibiting the expression of phosphorylated p65 within the NF-κB pathway, as well as downregulating both mRNA and protein expression of Keap1 in the Nrf2 pathway, thereby alleviating oxidative stress and endoplasmic reticulum stress in the kidneys, which may contribute to the amelioration of AKI symptoms. In target fishing, alisol B was used to directly target Gln384 hydrogen bond and soluble epoxy hydrolase for SPR experiment verification (Zhang et al., 2023). The research team from Sudan employed the independently developed Active-IT system to evaluate the efficacy of Ageratum Tigiatum against *Staphylococcus aureus*, *Escherichia coli*, *Pseudomonas aeruginosa*, and *Candida* albicans, utilizing thin-layer chromatography and HPLC for analysis (Sudan et al., 2021). Furthermore, the Shouhui Tongbian Capsule is recognized for its purgative effects, and is utilized in the treatment of chronic constipation. The magnetic microspheres of the total extract of Shouhui Tongbian capsule were used to obtain the target protein for high resolution mass spectrometry analysis. Target proteins were identified through a direct screening method known as target fishing, resulting in the selection of 138 target proteins and the enrichment of eight signaling pathways (Guo et al., 2021). Zhang et al. integrated two computational targeting techniques to identify 23 target proteins relevant to the treatment of cough with Beimu A (Zhang et al., 2020). The above content indicates that pharmacophore-based drug target screening can be applied to target fishing for natural products. This approach demonstrates superior efficacy in screening therapeutic targets for specific diseases using traditional Chinese medicine formulations or single herbal medicines, and can further elucidate their mechanisms of action.

### 2.1 Pharmacophore model

The pharmacophore model is a ligand-based methodology for the identification and screening of potential drug targets, offering significant advantages in the drug discovery process. Contemporary drug discovery often necessitates the screening of thousands of compounds; pharmacophore models facilitate the rapid evaluation of potentially active compounds on a large scale through computational simulations, thereby enhancing the efficiency of drug discovery efforts. These models can simulate the active conformations of ligand molecules through conformation searches and molecular superposition, allowing for the inference and elucidation of possible interaction patterns between receptor and ligand molecules. The identification of drug targets utilizing pharmacophore models can achieve a synergistic effect by targeting multiple sites (Sun et al., 2022). The pharmaceutical group model primarily employs pharmaceutical group matching servers for predictive analysis, including the CDD-CPI and DRAR-CPI servers (Ye et al., 2015). The pharmacophore model provides specific physicochemical properties, such as the number of hydrogen bonds, the quantity and distribution of hydrophilic and hydrophobic groups, and the number of aromatic rings. The number of databases available for screening based on pharmacophore models has been steadily increasing, with current databases including PharmaDB and HypoDB, among others. It is important to note that screening the same component across different databases does not guarantee the identification of identical targets. Typically, two databases are utilized in research: one for target identification and the other for validation, which helps to ascertain targets based on screening criteria such as molecular docking fit values and shape similarity (Lei et al., 2015). The core of this methodology lies in the selective validation of target proteins through a multi-angle scoring mechanism, which is essential for ensuring the reliability of the approach. However, a limitation of this method is that the scores generated by the scoring function do not necessarily correlate with the affinity of the compounds (Gao et al., 2013). Recursive partitioning, a multivariate data analysis technique that encompasses both single tree models and multi-tree bagged forests, has been integrated into pharmacophore models. This approach can rank active ingredients in natural products according to their activity levels, which may serve as a proxy for their affinity with target proteins (Zhang et al., 2018). Based on the pharmacophore model, a specialized automatic switching valve for HPLC was developed to enhance gene knockout or knock-in technologies in relation to gene function, thereby facilitating the identification of active ingredients in natural medicines (Song et al., 2016). In instances involving unknown biomolecules, the ADMET properties of natural drugs can be designed utilizing the BIOVIA Discovery Studio software package, which allows for the construction of the corresponding pharmacophore model (Tanwar et al., 2019).

Cyclin-dependent kinases (CDK), which function as core regulators of the cell cycle and transcriptional processes, have long been regarded as critical therapeutic targets in anticancer drug discovery due to their ability to drive uncontrolled tumor proliferation and sustain oncogene overexpression upon aberrant activation. However, first-generation pan-CDK inhibitors are often limited by their broad-spectrum inhibitory profiles, resulting in inadequate selectivity, narrow therapeutic windows, and significant systemic toxicity. In response, research efforts have shifted from “pan-CDK inhibition” toward a “precision-targeting” strategy, focusing on the development of next-generation highly selective CDK inhibitors designed to achieve safer and more effective therapeutic interventions against specific CDK family members. (Di Giovanni et al., 2016). Yau et al. integrated binding pose metadynamics with pharmaceutical agents to predict six targets associated with captopril, Lenvatinib, and a novel triazole-carboximidamide adenosine A2A receptor inhibitor. This approach enhanced the accuracy of molecular docking and the stability of target binding (Yau et al., 2024). The target protein CDK2 for curcumin was predicted through computational modeling. Following the preparation of curcumin-sepharose 4B beads, the fishing protein was validated via Western blotting experiments, ultimately demonstrating that curcumin directly binds to the ATP site of CDK2 (Lim et al., 2014). Clinical research has indicated that Marantodes pumilum exhibits therapeutic effects on breast cancer. Azfaralariff et al. selected five components of Marantodes pumilum for literature review and identified the top ten target proteins with the highest fitting scores using a reverse pharmacodynamic matching server. Through co-expression and gene analysis, the inhibitory effect of Marantodes pumilum on breast cancer cell proliferation was confirmed (Azfaralariff et al., 2022). Baruah constructed a library of active compounds derived from the natural product Leucaspera using the PubChem database and employed pharmacokinetic ADMET analysis to screen for drug-like components, further refining the targets through a reverse pharmacophore matching server (Baruah et al., 2022). A compound library of Aloe from Ethiopia was compiled, and its 3D structure was obtained from the PubChem database. The target of the compound was predicted using BIOVIA Discovery Studio based on a pharmacophore model (Bultum et al., 2022). A pharmacodynamic group model, grounded in 3D chemical characteristics, identified the targets of alkaloids in Maca, leading to the synthesis of eleven amide alkaloids, the screening of several pharmacological targets, and the confirmation of new therapeutic effects. Ultimately, models for prostate cancer, osteoporosis, and kidney diseases were selected to validate the predictive results of the targets, thereby affirming the reliability of the experimental findings (Yi et al., 2016). Glycopentalone, extracted from Glycosmis pentaphylla, has been reported to possess anti-hepatocellular carcinoma activity *in vitro*; however, the specific mechanism remains unclear. Targets were screened using a 3D pharmacophore database that included information on hydrophobic centers, positive centers, negative centers, hydrogen bond receptors, hydrogen bond donors, and aromatic rings, with the affinity sequence among the targets determined through docking experiments (Gurung et al., 2016).

Furthermore, the pharmacophore model exhibits significant flexibility and scalability. Researchers have the capability to modify the parameters and characteristics of the pharmacophore model to accommodate various compound libraries and target types, depending on specific research requirements. This adaptability enables pharmacophore models to be employed across a diverse array of drug development initiatives, encompassing both small molecules and biomacromolecules. The research team led by Jan Kihlberg investigated the modulation of binding pocket size during Keap1-Nrf2 binding by employing structural biological analysis in combination with computational chemistry and molecular dynamics simulations. Their work highlighted the dynamic nature of the binding site and the critical role of hydration networks, providing guiding implications for the development of novel therapies in fields such as cancer and neurodegenerative diseases (Begnini et al., 2022). Separately, Rojan Shrestha discussed the pharmacological characterization of binding interfaces using molecular dynamics simulations, specifically explaining the effects of solvation and conformational flexibility (Shrestha et al., 2021). With advancements in computational chemistry and bioinformatics technologies, the precision and applicability of pharmacophore models are continually enhancing. In comparison to traditional molecular docking methods, virtual screening based on pharmacophore models demonstrates superior efficacy in identifying the pharmacological targets of specific compounds, thereby providing a foundation for the subsequent identification of effective components (Yi et al., 2016).

### 2.2 QSAR model

The Quantitative Structure-Activity Relationship (QSAR) model is a method used to predict the biological activity of chemical compounds. By establishing a mathematical relationship between a compound’s structural characteristics and its biological activity, the QSAR model can aids researchers in identifying potentially active compounds. The basic principle of the QSAR model is to predict the biological activity of a compound based on its molecular structural attributes, which include physical and chemical properties, topological properties, and geometric characteristics. Through the analysis of the relationships between these structural features and biological activity, researchers can develop predictive models for the activity of novel compounds. The QSAR model can assess the interaction between ligands and targets using specific scoring programs, thereby providing robust support for the validity of the results. The Structure-Activity Relationship (SAR) model offers advantages such as low cost and high feasibility. A QSAR model utilizing a Random Forest (RF) algorithm has been established by the research team led by Kyoungyeul Lee, employing the Receiver Operating Characteristic (ROC) curve to evaluate the activity and inactivity of ligands for each target. The sample recall rate is utilized to validate the efficacy of this method (Lee et al., 2017). Building upon this foundation, researchers have integrated Support Vector Machine (SVM) techniques with Multiple Linear Regression (MLR) to further enhance the QSAR model, thereby improving the reliability of its predictive capabilities and addressing its limitations to a certain extent (Cao et al., 2012; Tur Razia et al., 2023). With the development of this method, Chakraborty et al. combined zebrafish animal models with QSAR models (Chakraborty et al., 2009). Linyan Zhu et al. also used QSAR model to study the toxicity of triclosan metabolites to 16 target proteins of zebrafish (Zhu et al., 2018).

Dipteris wallichii was extracted by different polar solvents, separated via TLC and HPLC, purified by column chromatography, get a new compound (E)-4-amino-1-(5-((1E, 4E)-hexa-1,4-dienyl)-1-methylpyrrolidin-2-yl) pent-2en-1-one. QSAR equation was obtained by Multi-Linear Regressions analysis and was compared with 34 known β-secretase-1 inhibitors to determine that the compound has therapeutic effect on Alzheimer’s disease (Chetia et al., 2020). At the same time, Subrata Das et al. used multiple linear regression QSAR to search for the active components of flavonoids in the A. anisophyllus of Acetylcholinesterase, a target protein of Alzheimer’s disease (Das et al., 2017). The human mitogen-activated protein kinase one was identified the target protein of N-Substituted Tetrahydro-β-Carboline Imidazolium Salt Derivatives by random Forest QSAR models, which provided theoretical basis for the discovery of anti-tumor drugs (Liang et al., 2017).

QSAR can be used for the preliminary screening of compounds. In large compound libraries, QSAR models can quickly identify compounds that may have target activity, thus reducing the effort and cost of experimental screening. In addition, QSAR model can be used to optimize compound structure. By analyzing the contribution of individual structural features to activity in the model, researchers can design and synthesize compounds with higher activity, and QSAR models can quickly predict the interaction between compounds and targets through computational methods, thus greatly improving screening efficiency, not only predicting the active ingredients of pharmaceuticals but also predicting the binding activity of known targets.

### 2.3 The mixed application of QSAR and pharmacophore

**FIGURE 3:**
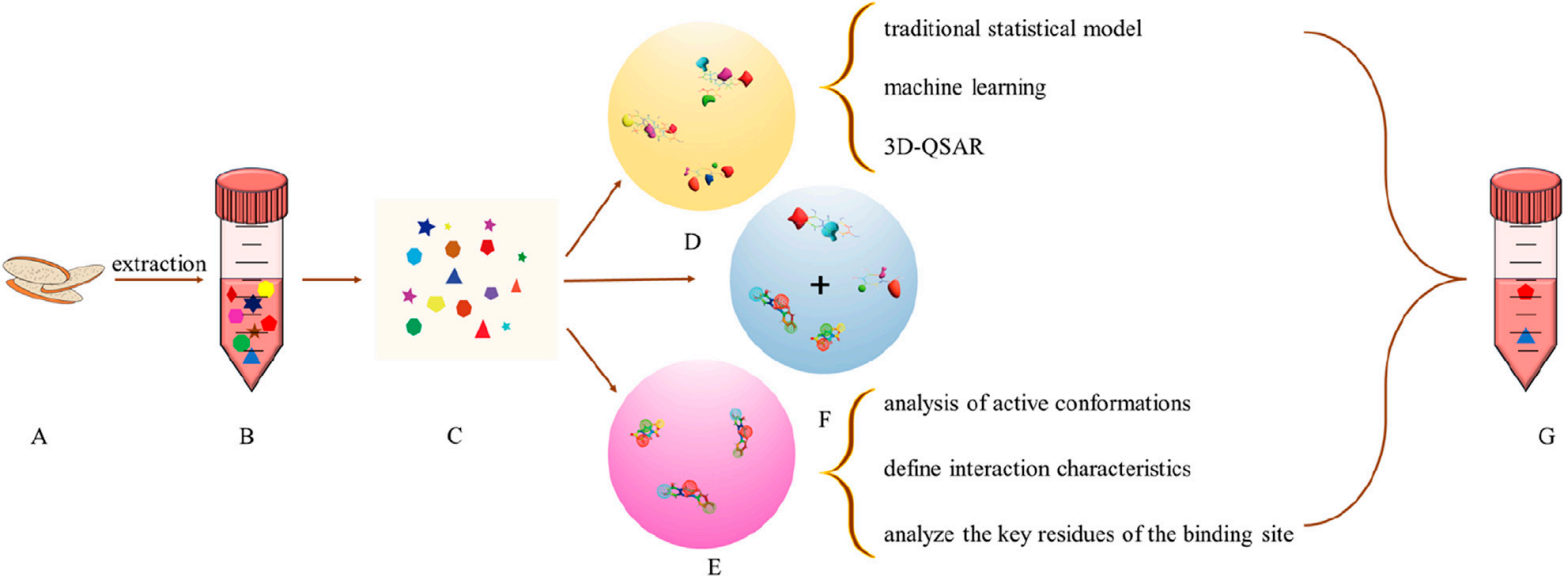
Ligand based target fishing **(A)** Natural Product; **(B)** natural extracts **(C)** Composition Database; **(D)** QSAR model; **(E)** pharmacophore model; **(F)** The combination of QSAR and pharmacophore model **(G)** Active Ingredient (The pharmacophore model is used for preliminary screening of compound libraries, rapidly narrowing down the range of candidate molecules, while the QSAR model can predict the activity and prioritize the screened compounds, optimizing candidate molecules)).

The above two methods do not necessarily use only one, can be combined to improve reliability (Figure 3). For example,: Xuewu Zhang et al. uses pharmacophore and 3D-QSAR to screen six tripeptide inhibitors of Dipeptidylpeptidase-4 VSM, ISW, VSW, ICY, ISD, and ISE(Wang, 2020). Reliability Evaluation of Drug Target Based on Ligand Method Based on Real-Time Update of Database and Analysis of Protein Structure and Biological Spectrum (Peón et al., 2016). In order to improve the accuracy of active ingredient search based on structural similarity, multiple databases will be used to search at the same time to compare the consistency of retrieval results so as to evaluate the rationality of the judgment method (Chávez-Fumagalli et al., 2018). Scoring function is an important evaluation index in structure-based molecular docking, including data-driven model and experience scoring function, whose introduction improves the accuracy of target fishing (Zheng et al., 2022). At the same time, it can also use multiple protein preparation software to simultaneously process proteins to prevent the deviation of experimental results caused by the contingency of single processing software (Chen et al., 2017). Ligand-Based targeting is generally targeted at ligands as small molecules, with few proteins or peptides as ligands, but the study has never stopped. The Chai team obtained paramyosins from marine animals, hydrolyzed the corresponding dipeptides using gastrointestinal digestive enzymes, and further predicted the anti-angiotensin-converting-enzyme dipeptides, which were then identified by molecular docking and molecular dynamics (Chai et al., 2022).

At present, the ligand-based target acquisition technology has gradually matured, but there are still many factors that cannot be overcome. Firstly, there is the active cliff phenomenon observed in the QSAR model (Stumpfe et al., 2019). For example, existing studies have found that Janus kinase 1 has a structure-activity relationship of the cliff structure, its efficacy and structure-activity relationship of research challenges (Daoud and Taha, 2020). When the target is screened according to the mechanism similarity, the potential information should be fully mined to reduce or avoid the activity “cliff” phenomenon (Bajorath, 2019). Secondly, the establishment of QSAR and pharmacophore relies on a large amount of preliminary basic data. Therefore, when there is limited basic data, the screening results may be inaccurate (Du et al., 2022). Pharmacophore is a spatial template that provides key functional groups. Therefore, when there is insufficient 3D structure of the target protein or high-quality ligand data, the construction of pharmacophore may deviate, resulting in false positives and false negatives during result analysis (Giordano et al., 2022).

To address biases in pharmacophore modeling arising from inadequate protein structural data or limited ligand information, a multi-level integrated strategy is proposed. Initially, a complementary framework combining AlphaFold2-predicted structures with ligand-based pharmacophore modeling should be established to mitigate constraints imposed by sparse initial data. Subsequently, molecular dynamics simulations and ensemble modeling approaches should be incorporated to enhance model robustness against conformational dynamics and data uncertainty. Most critically, a rigorous validation protocol encompassing decoy validation and independent test set evaluation must be implemented, forming a closed-loop optimization system of “computational prediction-experimental validation -model iteration.“This framework enables continuous refinement of pharmacophore parameters through feedback from *in vitro* activity data, systematically improving predictive reliability.

For addressing the complexity of multi-component and multi-target natural product research, it is essential to transcend traditional boundaries between computation, analysis, and experimentation by establishing a comprehensive research continuum. This integrated pathway can be summarized as: “network pharmacology for panoramic navigation → chromatography/mass spectrometry coupled with affinity-based fishing for precise compound identification → SPR/CETSA for interaction verification → gene editing techniques for functional confirmation. “This systematic approach enables breakthrough advancements from initial activity identification to comprehensive mechanistic elucidation.

Deep learning models, excelling at capturing complex nonlinear patterns, demonstrate higher predictive accuracy across both training and testing stages. Integrating them into QSAR frameworks can be regarded as a paradigm upgrade—one that not only elevates predictive performance but also propels the evolution of QSAR modeling architecture itself (Tropsha et al., 2024). Daniel Merk employed a variational autoencoder (VAE) as the core generative model, utilizing SMILES strings for molecular structure representation. By training an auxiliary predictive neural network, the VAE was guided to perform directed sampling in the latent space toward regions associated with ideal multi-target activity, thereby enabling the prediction of biological activity of corresponding molecules against multiple specific targets. This research methodology demonstrates a paradigm shift from the conventional “screening and optimization” approach to an “objective-driven automated creation” framework, highlighting the advanced capabilities of generative artificial intelligence in rational drug design (Isigkeit et al., 2024). The innovative aspect of diffPhore lies in its adoption of a denoising diffusion probabilistic model. This model employs a forward process to progressively add noise to an initially aligned ligand conformation, transforming it into a randomly unaligned state. Subsequently, through a reverse process, a neural network is utilized to incrementally remove the noise, thereby refining the conformation back into one that precisely matches the pharmacophore. Unlike conventional generative models, which rely primarily on data distribution, diffPhore incorporates external knowledge—such as molecular force fields—as strong constraints at each step of the reverse denoising process. This ensures that the generated results are not only statistically sound but also consistent with physicochemical principles (Yu J.-L. et al., 2025).

## 3 Receptor -based to screen drug targets

Target trapping based on receptor is to use the known target proteins to screen and predict the active components, and to verify the binding modes and mechanism of the proteins. This approach relies on the 3D structure of proteins, so the introduction of proteomics is very important for this approach. At present, homologous modeling is widely used. This method uses sequence homology of known structure to predict corresponding protein structure. If the two proteins have high enough sequence similarity, then their 3D structure is also more likely. Validity is the primary consideration in modeling, and a flexible hairpin shape loop that the binding pocket can be used to obtain a relatively open conformation by adjusting the size of the binding cavity according to the size of the ligand and the distance from the receptor (Sander et al., 2011).

The role of receptor structure analysis in drug target recognition is crucial. By analyzing the 3D structure of the protein, we can know how the protein binds to the potential drug molecule and choose the suitable binding pocket to identify the potential drug target. The core of the structural analysis of the receptor is the recognition of the protein active center, which is an important region of drug interaction. By locating these sites, the researchers looked for target compounds that could bind to them and regulate the function of proteins (ElGamacy and Van Meervelt, 2015). In the treatment of some diseases, the mutation of receptor may cause the change of drug binding site. By analyzing the structure of the mutant receptor, the problem of drug resistance can be solved (Gámez-Chiachio et al., 2022). Researchers can use the protein structure to design new drugs to overcome or reduce resistance, or to restructure existing resistant drugs to restore their effectiveness. This application is particularly important in the research of anticancer and anti-infective drugs.

**FIGURE 4:**
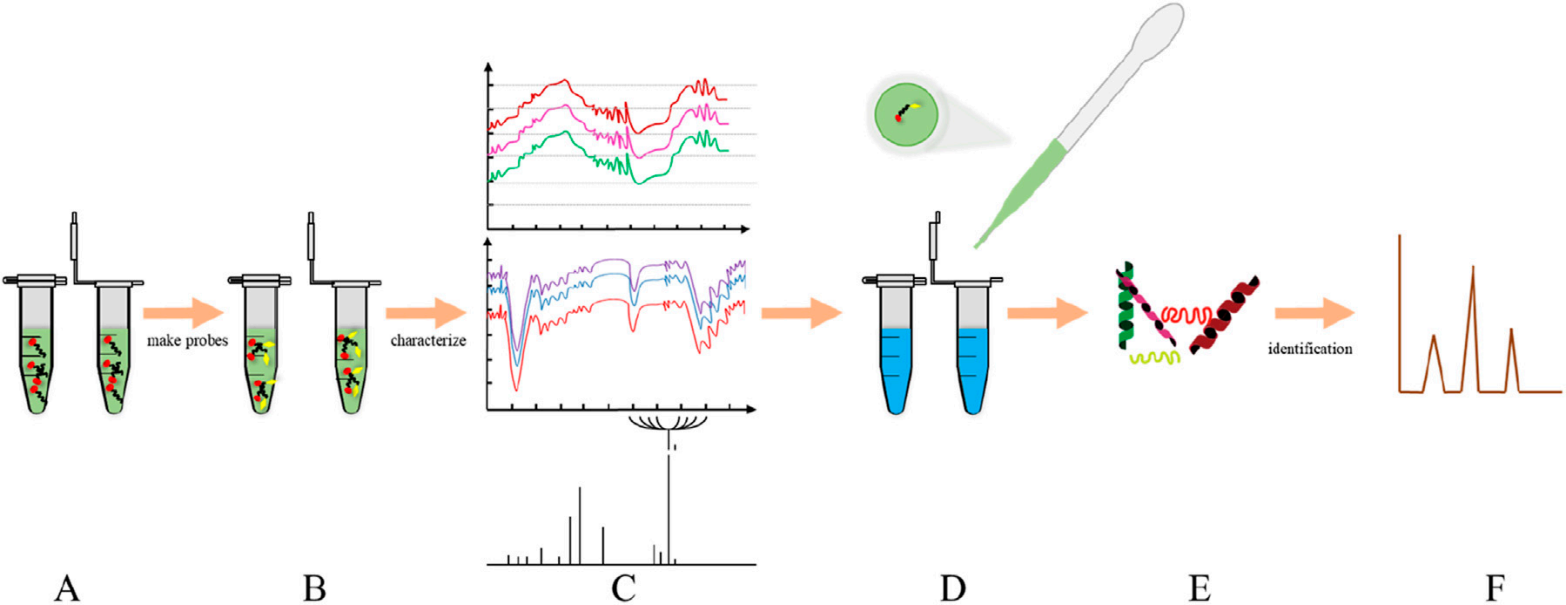
ABPP workflow: **(A)** Active molecule **(B)** protein probe; **(C)** characterization of protein probes; **(D)** cell lysate; **(E)** target protein; **(F)** Identification of proteins by MS.

Activity-based protein profiling (ABPP) is a method of directly questioning naturally expressed active proteins in highly complex biological environments (Xu et al., 2020). Compared with the traditional pulldown method, ABPP is a simple and efficient click-chemical method. After capturing the target protein, ABPP is analyzed by magnetic bead-pull mass spectrometry. The binding ability and morphology of protein and molecule were further verified by surface plasma and heat transfer. LC-MS is commonly used for protein identification by Click chemistry activity-based protein profiling (CC-ABPP) (Chen et al., 2020). This method is mainly to overcome the difficulty of protein probe entering cells. ABPP’s tandem orthogonal proteolysis technology (TOP-ABPP) is a fusion of click chemistry, biotin-streptavidin enrichment, which improves protein selectivity and general probe adaptability (Jiang et al., 2024). The core of the ABPP strategy is to design small molecular probes that can covalently interact with the target protein (Nodwell and Sieber, 2011). It is very important to design a protein probe with high specificity and selectivity for the successful experimental study. Thomas Bottcher’s team proposed a probe preparation method based on ligand selectivity (Schmid et al., 2022). Protein probes that use photoactive groups to make covalent bonds are the most widely used method. There are many photoaffinity groups, including Aryl Azide, Diazirine, Benzophenone and so on. There are two kinds of molecular probes. One is based on activity, the other is based on affinity (Geurink et al., 2011). Demonstrating its relationship to disease by inhibiting or activating protein activity is one of the main tools used in the study of natural products by the target fishing strategy (Figure 4).

Ping Li’s team developed a screening system to obtain sn-1,2-diacylglycerol inhibitor atractylenolide II and its direct target protein, DGKQ, for atractylenolide II in combination with the ABPP strategy. In addition, experiments showed that it could activate DGKQ-AMPK-PGC1a-UCP-1signaling in adipose tissue, providing evidence that atractylenolide II is a precursor for improving insulin resistance induced by obesity (Zheng et al., 2023). Using the same strategy, Sheng-Cai Lin’s team identified 113 target proteins using metformin probes coupled to their target proteins under ultraviolet radiation and then pulled down the conjugate with a neutral protein magnetic sphere (Ma, 2022). A small molecular probe of Bavachinin was designed and synthesized based on the CC-ABPP technique, and its target, Proliferating cell nuclear antigen (PCNA) was hooked. PCNA was identified by SPR, CETSA, DARTS and Co-IP test (Dong et al., 2024). Elisabeth Davioud-Charvet team used 3-benzyl-6-fluoromenadione AfBPP probe to catch a target against malaria parasites (Iacobucci et al., 2024).

In addition to ABPP, researchers have used other techniques to target fishing based on receptors, for example,: Dingqi Zhang screens the crystal of RBD of the spike protein SARS-CoV-2 from the PDB database, downloads the chemical structure from the PubChem database for molecular docking, and sorts the VS scores of the best binding poses according to the VS scores of the highest rank of active ingredients for subsequent validation experiments (Zhang et al., 2021). Rehman et al. isolated 14 compounds from *Lycium shawii* and *Aloe vera*, and found Carbonic anhydrases which is the effective target for cancer. 1,8-dihydroxy-3-(hydroxymethyl)anthracene-9,10-dione was obtained from inverse docking was the most effective component for cancer treatment (Ur Rehman et al., 2020). In cisplatin-induced deafness mice model, Tiliroside reduces apoptosis and oxidative stress and treats the cisplatin ototoxicity by deactivating the activity of aldose reductase (Liao et al., 2024). Spiro-acridine derivatives was used to reverse the virtual screening to find inhibitors of chitinase (De Oliveira Viana et al., 2023). Eight compounds of 4-Phenyl-1,3-Thiazole-2-Amines skeleton series were screened by reverse target to obtain the best target protein for treating leishmaniasis (Rodrigues et al., 2018). Synthesis of 8 3-aryl-4-alkylpyrazol-5-amines derivatives by target fishing to find the most effective anti-tumor components 5 h (Ma et al., 2020). Mitochondrial chaperonin HSP60 has been proved to be a directly targeted functional target protein of Myrtucommulone regulating apoptosis. In heat shock test, the LRP130 and LONP proteins regulated by HSP60 protein were proved to be indirect targets of MC inducing apoptosis (Wiechmann et al., 2017). Screening of real anticancer targets for Calactin, Calotropin, and Calotoxin to provide new ideas for the development of anticancer drugs (Parthasarathy et al., 2021). Leishmania donovani NH (LdNH) is a key target for the treatment of leishmaniasis. Extracts from M. oleifera leaves and flowers have therapeutic effects on Leishmaniasis. The bioactive compounds in the extracts were obtained through ligand fishing experiments. The specific components were identified by UPLC-MS, and their contents were directly determined by HPLC (De Faria et al., 2022).

ABPP, pharmacophore modeling, and QSAR represent complementary methodologies in drug discovery, spanning from experimental validation to theoretical prediction. ABPP, with its functional and global experimental capabilities, plays an indispensable role in target identification and validation. Pharmacophore modeling, leveraging its intuitive conceptual framework, demonstrates considerable utility in virtual screening and scaffold hopping. In contrast, QSAR capitalizes on its capacity for quantitative prediction, serving a critical function in the lead optimization phase. Consequently, the integration of these three techniques into a synergistic strategy—for instance, employing ABPP for target validation and initial lead discovery, followed by pharmacophore modeling for scaffold refinement and virtual screening, and finally utilizing QSAR models for precise activity prediction and optimization—holds significant potential to enhance the overall efficiency of the drug discovery process (Table 1).

**TABLE 1:** Comparison of the advantages and disadvantages of various target fishing.

	Pharmacophore	QSAR	ABPP
Data requirements	Active molecular structure information	Bulk structure-active data	Probe design and synthesis
Scope of application	Wide	Small molecule compound	Clear targets of enzymes and active sites
New target discovery	Limited	Limited	Strong
Experimental verification	Virtual filtering requires experimental verification	Experiment validation following prediction	Direct laboratory validation
Technology threshold	Medium	Low to high models	High
Cost and time	Low	Low	High

The use of Target fishing in drug discovery and development is increasing, and its use in multi-pharmacology could help find new targets for old drugs and expand the scope of clinical treatment. The same target for the same disease can be studied in different ways. Taking the targeted treatment of Alzheimer’s disease with ace methylcholinesterase (AChE) as an example: Anupam D. Talukdar’s team used Lipinski rule filtering, ADME/Tox screening, molecular docking and QSAR analysis, as well as *in vitro* activity evaluation to predict and confirm the inhibitory effect of flavonoids contained in Artocarpus anisophallus on AChE (Das et al., 2017). Jose Manuel Villalgordo et al. screened novel highly selective AChE inhibitors from compound libraries based on ligand based three-dimensional shape similarity and pharmacophore model screening, as well as structure based molecular docking and pharmacophore guided re scoring, using a drug repositioning strategy combined with a structure ligand dual drive design method (Pérez-Sánchez et al., 2021). In order to avoid the side effects generated during the treatment of AChE (acetylcholinesterase) inhibitors, the Haopeng Sun team also found but Butyrylcholinesterase inhibitors by combining pharmacophore construction with molecular dynamics simulations (Lu et al., 2019).

## 4 Methodology and discussion

The proteins obtained via target fishing can be systematically validated by multi - scale computational methods, including molecular dynamics, quantum chemical calculations, and molecular docking, to evaluate the biological rationality of the screened protein structures. Yi Mao employed the coarse-grained Elastic Network Model (ENM) to analyze the crystal structures of HIV-1 protease before and after binding (with substrate and nine FDA-approved inhibitors). The correlation matrix was used to describe the dynamic coupling between residues. Additionally, the Markov Clustering (MCL) algorithm was employed to identify functionally relevant residue networks, and the dynamic differences in residues between the bound and unbound states were compared (Mao, 2011). Through molecular dynamics simulations and free energy calculations, Xiaoyun Wu et al. systematically elucidated the binding mechanisms of EGFR allosteric inhibitors and their dynamic regulatory networks, revealing dynamic communication pathways between the allosteric site and the kinase functional domain. This work provides a theoretical foundation for designing highly selective allosteric drugs (Wu et al., 2022). Angus T. Voice et al. employed QM/MM simulations combined with molecular dynamics (MD) pre-equilibration methods to investigate the covalent bonding process between ibrutinib and Cys481 residue in BTK’s active site via Michael addition reaction. Their study delineated the atomic-level reaction pathway and energy profile, demonstrating the powerful capability of QM/MM methodology in deciphering covalent drug reaction mechanisms (Voice et al., 2021).

Integrating these multi-dimensional computational evidences, the research team not only validated the thermodynamic stability of protein conformations obtained through targeted conformational sampling, but also atomistically revealed the mechanism of ligand-induced allosteric effects. These findings establish a solid theoretical framework for subsequent structure-based drug design targeting this therapeutic target.

Natural products are characterized by multiple components and targets. Thus, when exerting pharmacological effects in organisms, natural products inevitably interact with other biological targets, resulting in toxic side effects. This has driven continuous research and clinical advancement in targeted drugs. Compared to traditional drugs, targeted drugs exhibit stronger specificity for particular cells while minimizing effects on normal cells. Particularly in cancer clinical treatment, targeted therapy demonstrates lower cellular toxicity and higher safety when compared to chemotherapy and immunotherapy. There are 15 FDA-approved targeted drugs in clinical use: Datopotamab Deruxtecan, Telisotuzumab Vedotin, Gemtuzumab ozogamicin, Brentuximab vedotin, Trastuzumab emtansine, Inotuzumab ozogamicin, Moxetumomab pasudotox, Polatuzumab vedotin, Enfortumab vedotin, Trastuzumab deruxtecan, Sacituzumab govitecan, Disitamab vedotin, Loncastuximab tesirine, Tisotumab vedotin, and Mirvetuximab soravtansin (Liu et al., 2024). Taking Polatuzumab vedotin as an example, it treats diffuse large B-cell lymphoma by inhibiting MCL-1 to induce apoptosis.

Research teams have employed an integrated multidisciplinary approach combining molecular docking technology, surface plasmon resonance (SPR) analysis, and bioactivity validation to systematically elucidate the structure-activity relationships (SAR) and mechanisms of action (MOA) of candidate compounds, thereby establishing critical theoretical foundations for clinical translation. For instance, the STAT3-selective inhibitor OPB-31121, currently undergoing Phase II clinical trials, has had its molecular recognition characteristics resolved through X-ray crystallography-based structural biological characterization (Huang et al., 2020). Furthermore, innovative applications of proteolysis-targeting chimera (PROTAC) technology to investigate targeted degradation mechanisms of the clinical-stage STAT3 inhibitor napabucasin have provided novel insights for addressing drug resistance challenges (Hanafi et al., 2021). Notably, the lymphocyte-derived agent (LDA), recognized as the first-in-class small-molecule inhibitor targeting tumor necrosis factor receptor-associated factor 2 (TRAF2), has demonstrated significant antitumor efficacy in preclinical models along with favorable pharmacokinetic properties, demonstrating promising clinical translation potential (Yan et al., 2022). These representative cases underscore the expanding applicability of computational target identification methodologies in contemporary drug discovery pipelines.

In recent years, with the development of technologies such as X-ray crystallography (Zha et al., 2016), bioinformatics (Li et al., 2019), NMR spectroscopy (Ur Rehman et al., 2020) and freezing electron microscope (Wang et al., 2024), significant progress has been made in the structural analysis of receptors. The analysis of receptor structure can help identify drug targets and provide important information for drug design. This structure- oriented CAD improves the accuracy of drug selection, reduces the possibility of side effects, and reveals the relationship between receptor conformation and mechanisms of action.

The introduction of computer and molecular dynamics simulations provides support for researchers to model receptors and their interactions between receptors and drug molecules at the molecular level. With the introduction of machine learning and artificial intelligence technology, the predictive power and accuracy of virtual filtering have improved significantly (Carullo et al., 2023). For example, the combination of intelligent recognition technology and mass spectrometry was used to analyze the components of Xiaokewan, and computer simulation docking and network pharmacology were used to screen the anti-diabetic activity of the high-exposure components of Xiaokewan in the mass spectrum, and the efficacy experiment was verified (Zhu et al., 2020). By training machine learning models, researchers can more accurately predict the activity and selectivity of compounds, thus improving the success rate of virtual screening (Bagherian et al., 2021).

In recent years, artificial intelligence, machine learning, and biosensors have emerged as hot topics, and their applications in target identification have gradually deepened, continuously providing new technologies and possibilities for the advancement of this field. Over the past 5 years, artificial intelligence has rapidly integrated into various industries, including the pharmaceutical sector. In drug discovery, AI-driven target identification and synthesis planning have become pivotal (Zhang, n.d.). Particularly in oncology drug target identification, artificial intelligence technologies not only facilitate the discovery of novel anti-tumor targets but also efficiently record and quantify interactions among various components in cancers and other diseases, laying the foundation for developing new anti-tumor drugs (You et al., 2022). Firstly, network-based biological analysis algorithms offer alternative pathways for identifying cancer targets. Secondly, machine learning-driven bioanalytical methods effectively process high-throughput, heterogeneous, and complex molecular data, extracting intricate biological networks. For instance, Diego Galeano’s team developed the “sChemNET” machine learning approach and applied it to miRNA target discovery (Galeano et al., 2024). The high precision, sensitivity, and real-time monitoring capabilities of novel biosensors are increasingly valued in drug target discovery. These biosensors play a critical role in conformational dynamics and biological signal transduction, aiding in the detection of downstream protein signals in signaling pathways and elucidating the conformational relationships between drugs and their targets. This provides deeper theoretical insights into drug mechanisms. For example, G protein-coupled receptors (GPCRs), a superfamily of transmembrane signaling proteins that mediate chemical signal transduction across membranes, are ideal drug targets. Research on GPCRs biosensors has long been a focus and has achieved significant progress (Saca et al., 2025). Fluorescent and bioluminescent biosensors are crucial in targeted drug discovery for oncology (Kelly and Yang, 2024), while redox protein-based fluorescent biosensors have been successfully developed and applied to study the anti-inflammatory mechanisms controlled by Msr B1 (Shim et al., 2024).

## 5 Conclusion

To effectively address the core challenges of data quality, model generalization, and validation reliability faced by pharmacophore and QSAR models in natural product research, a multidimensional synergistic strategy is essential. This can be achieved by integrating multi-source data—including AlphaFold2-predicted structures and chemical proteomics—to enhance fundamental data quality; employing advanced algorithms such as graph neural networks and multi-task learning to improve model characterization of complex chemical spaces; establishing a closed-loop optimization workflow of “computational prediction-experimental validation-model iteration” utilizing orthogonal verification techniques like SPR and CETSA; and combining reverse target fishing with network pharmacology to systematically analyze the multi-component, multi-target interaction networks of natural products. This integrated paradigm, synthesizing data, algorithms, validation, and systems biology perspectives, will significantly enhance the accuracy of active ingredient identification and the depth of mechanistic interpretation.

A large number of studies have shown the feasibility of target fishing in drug design. At present, the application of target fishing technology in drug design is still combined with computer-aided drug design. It is still a big challenge to evaluate the rationality of drug design for computer-aided drug involving 5V characterization, namely, volume, velocity, variety, variability, veracity (Zloh and Kirton, 2018). Based on ligand-based active component screening often encounters the challenge of activity cliffs (ACs). To improve prediction accuracy, existing studies have adopted MMP models based on specific chemical substructures to replace traditional computational similarity metrics, thereby relying on statistical evidence from large-scale experimental data rather than theoretical speculation (Hu et al., 2012). The ACtriplet deep learning model, by integrating a triplet loss function and pre-training techniques, effectively enhances the ability to identify key subtle structural differences that trigger Acs (Yu X. et al., 2025). How to more accurately predict and interpret Acs remains an important direction worthy of further attention in future research.

Despite its existing limitations, target fishing technology has demonstrated significant potential in the field of drug screening, particularly in natural medicine research. Given the complexity of natural medicines characterized by multi-component and multi-target profiles, the continued development and optimization of this technology are of paramount importance. To advance the standardization and practical application of target fishing technology, future research should focus on the following dimensions: establishing standardized benchmark datasets covering diverse target types and activity levels to provide a unified framework for algorithm evaluation; deeply integrating three-dimensional structural data from structural proteomics and cryo-electron microscopy with artificial intelligence technologies to develop dynamic binding site prediction models; and refining standardized workflows from computational prediction to experimental verification by constructing multi-level validation systems utilizing technologies such as surface plasmon resonance and chemical proteomics. Through systematic advancement in algorithm benchmarking, data integration, and validation pipeline optimization, the reliability and translational value of this technology in natural medicine research will be significantly enhanced. Furthermore, promoting interdisciplinary applications of target fishing technology is crucial, requiring the integration of multidisciplinary knowledge to establish comprehensive research methodologies (Lima et al., 2021). Such cross-disciplinary approaches will facilitate a comprehensive understanding of the mechanisms of action of active constituents and provide innovative perspectives for new drug development.
